# Long-Term Observation of Mixing States and Sources of Vanadium-Containing Single Particles from 2020 to 2021 in Guangzhou, China

**DOI:** 10.3390/toxics11040339

**Published:** 2023-03-31

**Authors:** Xin Xiong, Zaihua Wang, Chunlei Cheng, Mei Li, Lijun Yun, Sulin Liu, Liyuan Mao, Zhen Zhou

**Affiliations:** 1 Institute of Mass Spectrometry and Atmospheric Environment, Guangdong Provincial Engineering Research Center for Online Source Apportionment System of Air Pollution, Jinan University, Guangzhou 510632, China; 2 State Key Laboratory of Organic Geochemistry, Guangzhou Institute of Geochemistry, Chinese Academy of Sciences, Guangzhou 510640, China; 3 Institute of Resources Utilization and Rare Earth Development, Guangdong Academy of Sciences, Guangzhou 510650, China; 4 State Key Laboratory of Loess and Quaternary Geology, Institute of Earth Environment, Chinese Academy Science, Xi’an 710061, China; 5 Guangdong-Hongkong-Macau Joint Laboratory of Collaborative Innovation for Environmental Quality, Guangzhou 510632, China

**Keywords:** vanadium, single particle, ship emissions, long-term observations, source apportionment

## Abstract

The distribution of vanadium (V) in aerosols is commonly used to track ship exhaust emissions, yet the atmospheric abundance of V has been greatly reduced due to the implementation of a clean fuel policy. Recent research mainly discussed the chemical compositions of ship-related particles during specific events, yet few studies focus on the long-term changes of V in the atmosphere. In this study, a single-particle aerosol mass spectrometer was used to measure V-containing particles from 2020 to 2021 in Huangpu Port in Guangzhou, China. The long-term trend of the particle counts of V-containing particles declined annually, but the relative abundance of V-containing particles in the total single particles increased in summer due to the influence of ship emissions. Positive matrix factorization revealed that in June and July 2020, 35.7% of the V-containing particles were from ship emissions, followed by dust and industrial emissions. Furthermore, more than 80% of the V-containing particles were found mixing with sulfate and 60% of the V-containing particles were found mixing with nitrate, suggesting that the majority of the V-containing particles were secondary particles processed during the transport of ship emissions to urban areas. Compared with the small changes in the relative abundance of sulfate in the V-containing particles, the relative abundance of nitrate exhibited clear seasonal variations, with a high abundance in winter. This may have been due to the increased production of nitrate from high concentrations of precursors and a suitable chemical environment. For the first time, the long-term trends of V-containing particles in two years are investigated to demonstrate changes in their mixing states and sources after the clean fuel policy, and to suggest the cautious application of V as an indicator of ship emissions.

## 1. Introduction

The vanadium (V) in atmospheric particles originates from a variety of natural and anthropogenic sources, including continental dust, marine aerosols, fossil fuels, biomass burning, and vehicle exhaust [1,2,3,4,5,6,7,8]. V can induce lung tumors and has neurotoxic effects on the nervous system, and it is a non-carcinogenic heavy metal [9,10,11,12,13]. The mass loading of V in PM_2.5_ varies by region, and the concentration is usually higher in coastal and port areas. According to previous studies, the concentrations of V in Shanghai Chongming Island (5.69 ng/m^3^) [14], Meishan Port (4.3 ng/m^3^) [15], Pudong (4.6 ng/m^3^) [16], Xiamen (10.6 ng/m^3^) [17], and Hong Kong (15.7 ng/m^3^) [18] were higher than those in urban areas such as the urban region of Guangzhou (3.4 ng/m^3^) [19], the suburban region of Guangzhou (1.95 ng/m^3^) [19], and the urban region of Beijing (0.66 ng/m^3^) [20].

In recent years, the distribution and characteristics of V in ship-emitted particles have attracted a great deal of attention. High loadings of V can be emitted from the combustion of heavy fuel oil in ship engines, and thus, the presence of V has generally been regarded as an effective tracer of ship emissions [20,21,22,23,24,25,26]. Since China designated three domestic emission control zones (DECAs) in 2015, i.e., the Yangtze River Delta (YRD), Pearl River Delta (PRD), and Bohai Rim Area (BRA), the quality of ship fuel oil has been forced to improve to meet the demand for cleaner ship emissions. All vessels inside the DECAs were required to use fuel oil with a sulfur content of less than 0.5% after 1 January 2020 [20], which also helped to reduce the content of V in ship fuel oil. After the implementation of this clean fuel policy, the V content of emissions decreased significantly. A recent study conducted in Pudong [16] revealed that the average concentration of V was 1.19 ng/m^3^ in 2020, which was 74% lower than that in 2019 (4.60 ng/m^3^).

In addition to the offline determination of the mass concentration of V in particles, the distribution and mixing states of V in ship emission-related single particles have also been studied. Single-particle aerosol mass spectrometry (SPAMS) has been widely used to identify the temporal and spatial distributions of V in single particles due to the precise ionization and detection of V of this method [27,28,29,30]. The presence and mixing states of V in coastal areas are mainly affected by ship emissions, industrial processes, and regional transportation [28,31,32,33,34,35]. According to previous studies using SPAMS, ship-emitted particles are dominated by elemental (EC) and organic carbon, V, sulfate, and nitrate, which have stronger signals than ambient particulates [22,28,30,36,37,38,39]. Another study of single particles in ship plumes in Shanghai Port found that the freshly emitted ship particles were dominated by sulfate, EC, and V, but they exhibited a very low nitrate signal or no nitrate signal [28]. Sulfate is known to be formed rapidly in ship stacks from the gas–particle conversion of SO_2_, and Xiao et al. (2018) found that sulfate particles in the ambient air always remain synchronous with ship-source particles, which confirmed the relatively stable contribution of ship-source sulfate particles to the ambient sulfate particle concentration [30,39]. In addition, the abundance of sulfuric acid in the V-containing particles is higher than that in particles that do not contain V, suggesting that V may act as a catalyst during the oxidation of SO_2_ to sulfate in these particles [28,36,40]. After the implementation of the clean fuel policy, field observations in Shanghai and Guangzhou showed that V was still prevalent in particles from ship plumes, but the V content was significantly reduced [16,28,29,41]. These studies found that V-containing particles were still closely related to ship emissions; however, after the implementation of the clean fuel policy and the outbreak of the COVID-19 epidemic, the distribution and sources of the V-containing particles may have changed. Hence, a comprehensive investigation of these particles is necessary to understand their sources and aging processes.

To investigate the long-term trend of the mixing state of the V-containing particles, V-containing single particles were continuously determined via SPAMS in 2020 and 2021 in Huangpu Port, Guangzhou. The chemical compositions and mixing states of these particles in the different seasons were compared. In the first part of this paper, the necessity to conduct the long-term study of V-containing single particles and the novelty of this work were described; in the second part, the single particle collection and data processing were presented, as well as the sources analysis via positive matrix factorization (PMF) and potential source contribution function (PSCF); in the third part, the annual variation and mixing states of V particles were discussed, and their sources and aging processes were investigated. In addition, the long-term trends of the mixing states of V with sulfate and nitrate were investigated to evaluate the changes in ship emission sources and the aging state of the V-containing particles. The main conclusions, limitations of this work, and future directions were summarized in part four. The results of this study provide insights into the impact of the clean fuel policy on the distribution of V and its role as a tracer for ship emissions.

## 2. Materials and Methods

### 2.1. Sampling Information and SPAMS Measurements

**Figure 1:**
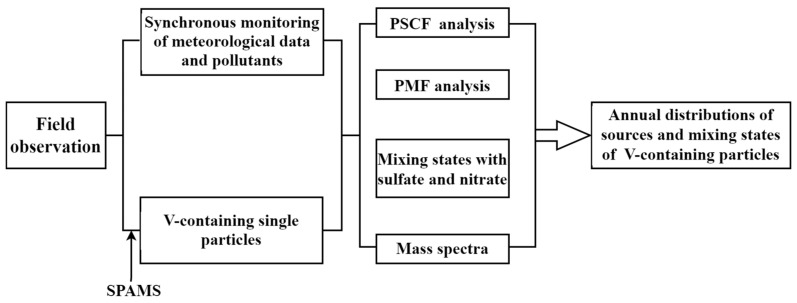
The flow chart of sampling and analytical methods.

Ambient single particles were measured via SPAMS in Huangpu Port, Guangzhou, China, from 1 January 2020 to 31 December 2021. The sampling site was located on the northern coast of the Pearl River. Huangpu Port is the largest coastal and ocean transportation hub in southern China. The hourly concentrations of PM_2.5_, PM_10_, and gaseous pollutants, as well as meteorological data, were simultaneously collected. The sampling methods and data analysis used in this work are shown in Figure 1.

The SPAMS was designed by the Guangzhou Hexin Analytical Instruments Limited Company based on the same principle as the aerosol time-of-flight mass spectrometer (ATOFMS) developed by Prather and co-workers [42,43,44]. The particle detection method of SPAMS has been described in detail by Li et al. (2011) [45]. Briefly, aerosol particles are introduced into the SPAMS through the orifice and are accelerated through an aerodynamic lens into the sizing region of the instrument, and are identified based on how fast they pass through two continuous diodes (Nd:YAG) and a 532 nm laser beam in the sizing region. The sized particles are then ionized by a 266 nm Nd:YAG laser (0.6 mJ), which is triggered at the precise time determined by the particle velocity. The positive and negative ions were analyzed using a Z-bipolar time-of-flight mass spectrometer [45,46,47].

### 2.2. Data Analysis

The chemical compositions and mixing states of the single particles measured via SPAMS were analyzed using the Computational Continuation Core (COCO) toolkit in MATLAB. In this study, a total of 37,867,836 particles were detected in two years. The V-containing particles were identified by the marker ions of ^51^V^+^ and ^67^VO^+^ with a relative peak area (RPA) threshold greater than 0.1% of the total signal in the mass spectrum [29,35,40]. According to this principle, the particle count of the V-containing particles was 373,805, which accounted for approximately 1% of the total particles in two years. In this paper, the definition of the mixing state based on SPAMS refers to the coexistence of two or more chemical components in the same particle, which can be used to track the origin and chemical evolution processes of specific components [48,49,50,51].

To implicitly track the chemical species that co-varied with V in the single particles, PMF was conducted to resolve the different types of V-containing particles. Similar analytical methods have been used in studies of the evolution of biomass burning emissions using the single-particle mass spectrometry technique [52,53]. Twenty-two marker ions were selected to conduct the PMF analysis, comprising elemental carbon (^24^C_2_^+^ and ^36^C_3_^+^), organic carbon (^27^C_2_H_3_^+^, ^37^C_3_H_7_^+^, ^41^C_3_H_3_^+^, and ^43^C_3_H_7_^+^), metals (^23^Na^+^, ^40^Ca^+^, ^51^V^+^, ^52^Cr^+^, ^55^Mn^+^, ^58^Ni^+^, and ^63^Cu^+^), organic nitrogen (^26^CN^−^ and ^42^CNO^−^), oxidized organics (^59^CH_3_CO_2_^−^, ^71^C_3_H_3_O_2_^−^, and ^73^C_2_HO_3_^−^), ^56^CaO^+^, sulfate (^97^HSO_4_^−^), nitrate (^62^NO_3_^−^), and ^35^Cl^−^. These components were abundant in the V-containing particles and were usually associated with specific sources and/or secondary processes. According to previous studies that used PMF to analyze single particles, the uncertainty was defined as 50% of the relative peak area of the marker ion [52,53], and two to seven factors were generated through PMF analysis of the V-containing particles. Based on their strong factor information and the moderate correlation of the RPAs under measurement and prediction, seven factors were selected as the best solution for the determination of the V-containing particles.

### 2.3. Potential Source Contribution Function Analysis

The PSCF analysis is an effective method for identifying source-contaminated areas in recipient sites [54,55]. In this study, the PSCF was used to calculate the 72 h backward trajectory 500 m above the sampling site [56,57]. Briefly, the $ij_{th}$ component of a PSCF field can be expressed as follows:

$$PSCF_{ij}=\frac{m_{ij}}{n_{ij}},$$

where $n_{ij}$ is the total number of endpoints that fall in the $ij_{th}$ cell (divided into 0.5° × 0.5° latitude and longitude grids), and $m_{ij}$ is the number of endpoints of that parcel for which the measured values exceed a user-determined threshold criterion. Since the deviation of the PSCF results will increase with increasing cell–value receptor distance, to reduce the influence of the uncertainty of cells with small $m_{ij}$ values, the value of the PSCF is multiplied by the weighting factor $W_{ij}$ (hereinafter referred to as the WPSCF) [57,58]. The source distribution characteristics of V particles were similar between the three months of each season, and we have listed the PSCF results of March, April, and May in the Supplementary Materials (Figure S1). Thus, we have chosen the results of January, April, July, and October to represent the annual results of the PSCF analysis of the V particles.

## 3. Results and Discussion

### 3.1. Characteristics of V-Containing Single Particles

**Figure 2:**
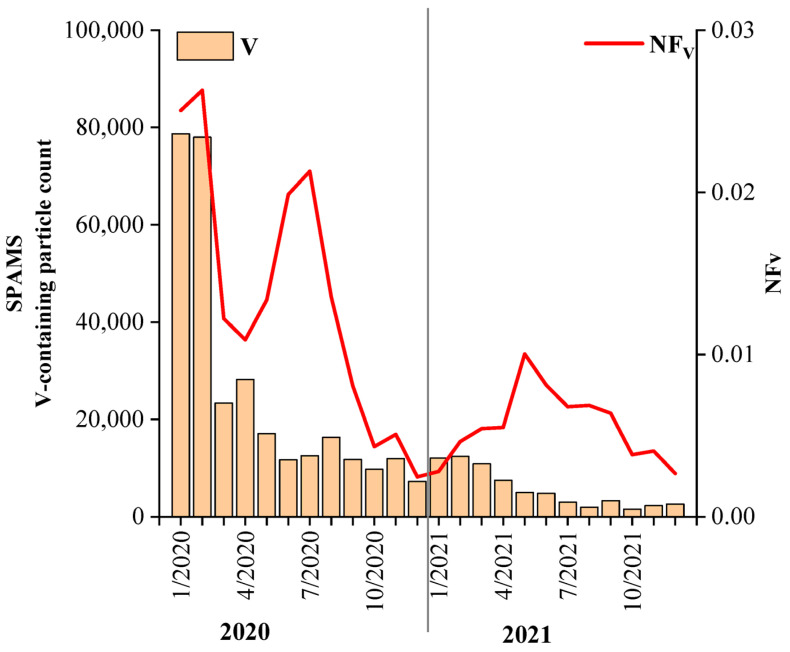
Variation trend of the number of V-containing particles and their proportions of the total particles in each month measured via SPAMS in Guangzhou, China, from 2020 to 2021.

The monthly variation trends of the V-containing particles based on the average of the hourly data are shown in Figure 2. The particle count of the V-containing particles accounted for 1.4% and 0.6% of the total detected single particles in 2020 and 2021, respectively. The particle counts of the V-containing particles exhibited a decreasing trend from the beginning of 2020 to 2021, which may have been due to two reasons. First, the quality of the ship fuel oil was improved according to the clean fuel policy, so the fuel oil contained a smaller amount of V. This led to a reduction in the V emitted from the ship exhaust. The second possible reason is the influence of the COVID-19 epidemic since January 2020. Many field studies have evaluated the impact of the epidemic on anthropogenic activities [59,60], and they reported a sharp reduction in primary anthropogenic emissions. Although the number fraction (NF) of the V-containing particles (NFv) exhibited a general decreasing trend from 2020 to 2021, the NFv exhibited peaks in May, June, and July. Furthermore, the NFv accounted for 2.1% of the total particles in July 2020 and 1.0% in May 2021. This phenomenon was primarily due to the summer monsoon transporting the air pollutants emitted by ships to the sampling site, which was observed in our previous study [35]. Other studies have also reported the predominant contribution of V from ship exhaust in the sampling site. For example, the concentration of V was higher in the southeastern coastal area and the Qingdao area during the summer [54,61]. In Xiamen, the monthly pattern of V concentration increased from April to September and then decreased from October to January [17].

**Figure 3:**
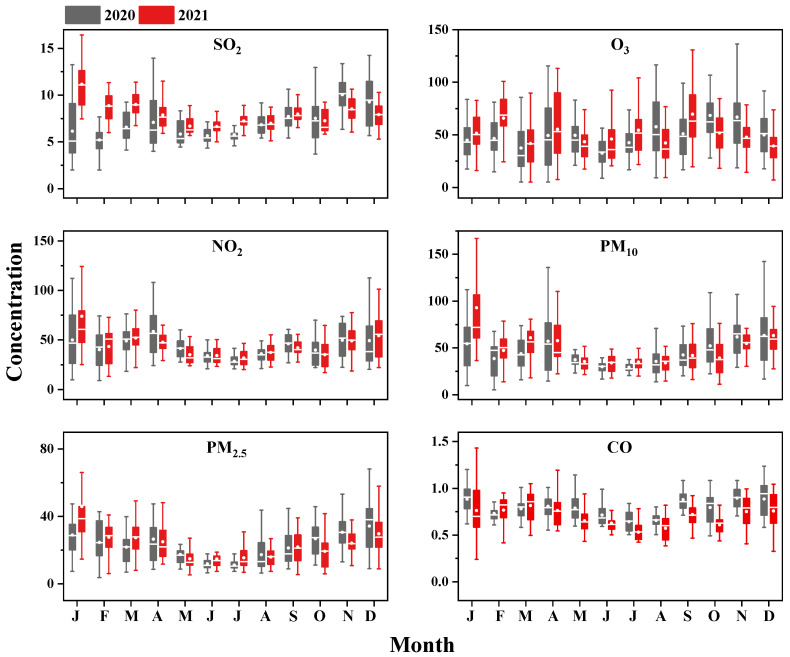
Boxplots of the monthly mean changes in the concentrations of six types of pollutants, SO_2_, O_3_, NO_2_, PM_10_, CO, and PM_2.5_, from 2020 to 2021. The units of the CO concentration are mg/m^3^, and the units of the concentrations of the other pollutants are μg/m^3^. The black boxes represent 2020 and the red boxes represent 2021.

The monthly variations in SO_2_, O_3_, NO_2_, PM_10_, CO, and PM_2.5_ concentrations are presented in Figure 3. All of these compounds exhibited trends different from that of the V-containing particles. The PM_2.5_ and PM_10_ concentrations were both high in winter and low in summer, which was mainly due to the different loadings of particulate matter (PM) from local anthropogenic emissions and transportation by the northerly wind in the different seasons. Figure S2 shows the temporal variations of meteorological parameters (wind speed, wind direction, temperature, and relative humidity), and the north wind prevailed in the winter, which brought large amounts of anthropogenic pollutants to Guangzhou. The CO, SO_2_, and NO_2_ concentrations were all high in the winter seasons and low in the summer seasons of 2020 and 2021, and they were mainly influenced by local emissions, including vehicle exhaust and coal burning. Although CO, SO_2_, and NO_2_ can all be produced from ship exhaust, the total amount of their emissions was far less than that of fossil fuel and biomass burning sources in urban cities [24,62]. Thus, the general trends of the CO, SO_2_, and NO_2_ concentrations were different from that of the V-containing particles from 2020 to 2021. As a secondary pollutant, O_3_ is produced from photochemical reactions of NOx and volatile organic compounds (VOCs) [63,64]. The O_3_ concentration peaks occurred in April, August, September, October, and November, which was completely different from the trend of the PM and primary gas pollutants.

**Figure 4:**
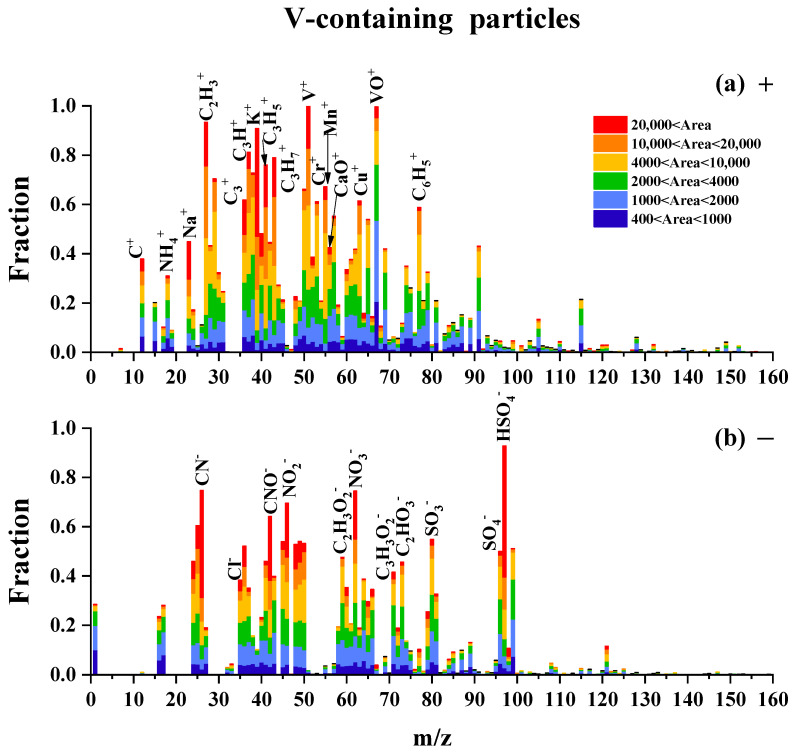
Mass spectra of V single particles. The color bars represent the peak areas corresponding to a specific ion in the single particles.

The average mass spectra of the V-containing particles in the summers of 2020 and 2021 were similar, so we selected the spectra of the V-containing particles in June and July 2020 for a detailed description (Figure 4). The positive mass spectrum was characterized by carbon clusters such as ^24^C^+^, ^36^C^+^, ^27^C_2_H_3_^+^, ^37^C_3_H_7_^+^, ^41^C_3_H_3_^+^, and ^43^C_3_H_7_^+^, and metal ions such as ^23^Na^+^, ^51^V^+^, ^52^Cr^+^, ^55^Mn^+^, ^58^Ni^+^, and ^63^Cu^+^. Meanwhile, the negative spectrum mainly contained sulfate (^97^HSO_4_^−^), nitrate (^62^NO_3_^−^), and oxygenated organics (^59^CH_3_CO_2_^−^, ^71^C_3_H_3_O_2_^−^, and ^73^C_2_HO_3_^−^). The mixing states of V with metals and secondary species suggest that these single particles were secondary particles.

### 3.2. Sources and Formation Processes of the V-Containing Particles

**Figure 5:**
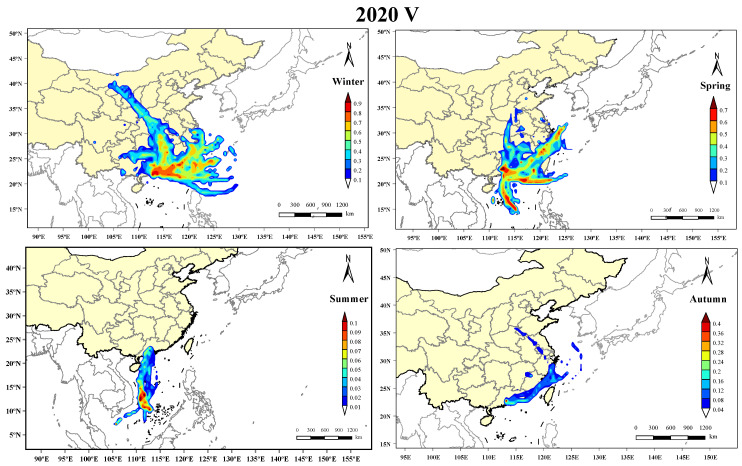
Potential source distribution of the particulate matter containing V in the four seasons in 2020. The hourly concentration of the particulate matter containing V was chosen as the standard threshold in the analysis. The colors in the legend represent the WPSCF values.

In this study, the PSCF analysis was conducted using the changes in the hourly particle counts of the V-containing particles. We chose the data from 2020 to conduct the investigation since the patterns of the 2020 and 2021 data for all four seasons were similar. The PSCF results (Figure 5) clearly show that the V-containing particles were associated with the transportation of different air masses in the four seasons. Regional and long-range transport both contributed significantly to the distribution of the V-containing particles in Guangzhou in winter, spring, and summer, which was attributed to the influence of ship emissions and oil refineries [14,54]. However, the transmission routes of the V-containing particles to the sampling site were different in these three seasons. In summer, the air masses were solely from the South China Sea, indicating the direct contribution of ship emissions to the distribution of V in Guangzhou, whereas in spring, the air masses were also from the east and northeast coastline, except from the South China Sea. In winter, although the air masses were still from the marine areas to the east, due to the low transmission rate, the majority of the V was from the region of the sampling site. This suggests the contribution of industrial emissions of V, including oil refineries and metal factories.

**Figure 6:**
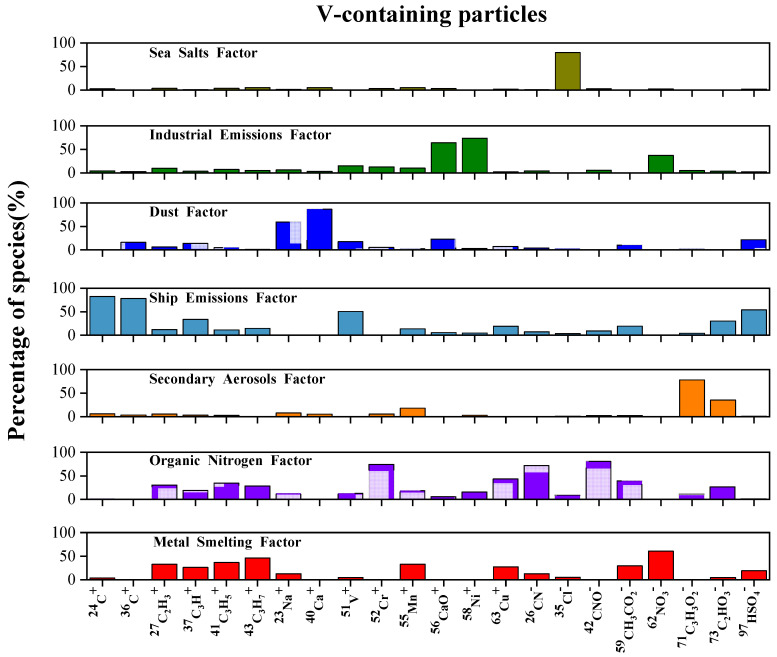
The PMF-resolved factors and their relative contributions to the predicted V-containing particles.

**Figure 7:**
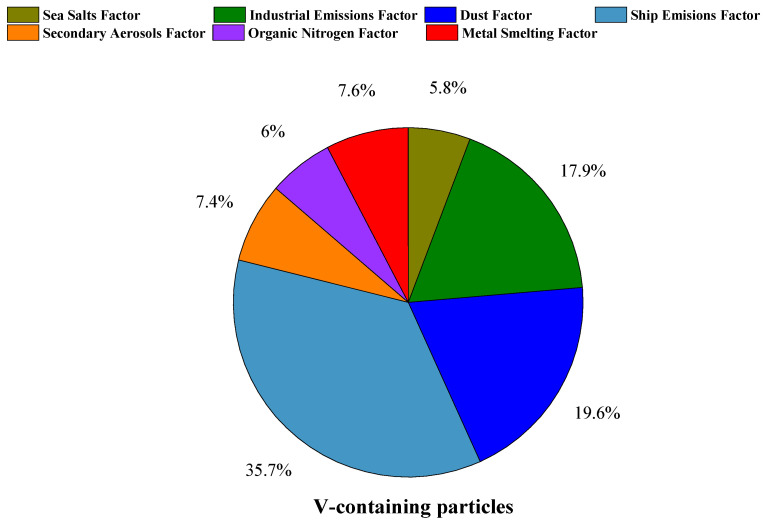
The fractions of the PMF factors in the V-containing particles.

To further explore the sources and influencing factors of the V-containing particles, we analyzed the data from June and July 2020. The seven-factor PMF profiles are presented in Figure 6. Based on the abundant markers and significant features of each factor, we named these factors separately. Seven factors of the V-containing particles were identified: a ship emissions factor, a dust factor, an industrial emissions factor, a metal smelting factor, a secondary aerosols factor, an organic nitrogen factor, and a sea salt factor. The ship emissions factor was composed of abundant ^24^C^+^, ^36^C^+^, ^51^V^+^, and ^97^HSO_4_^−^, which were significant in the particles emitted by ships [23,29,37,40]. Additionally, moderate amounts of ^46^NO_3_^−^ occurred in this factor, suggesting the aging of the V-containing particles during transport [36,38,40]. In the dust factor, ^40^Ca^+^ was the most abundant, indicating the contribution of suspended dust [8]. The industrial emissions factor and the metal smelting factor were both composed of nitrate, indicating that the particles associated with these sources were produced through atmospheric aging processes. According to previous studies, in addition to the combustion of fossil fuels, V-containing particles in Guangzhou could be from glass factories and ceramics factories [1]. In this study, the industrial emissions factor mainly consisted of ^56^CaO^+^ and ^58^Ni^+^, suggesting the influence of factory emissions in addition to an oil combustion source [21,65]. Furthermore, Cu is a typical tracer of the steel industry. In this study, the metal smelting factor was composed of moderately abundant ^65^Cu^+^, implying that a certain amount of the V was connected with metal manufacturing [65]. The secondary aerosols factor mainly comprised secondary ions, including ^71^C_3_H_3_O_2_^−^ and ^73^C_2_HO_3_^−^. The organic nitrogen factor was composed of abundant ^42^CNO^−^, ^26^CN^−^, and ^59^CH_3_CO_2_^−^, indicating the aged state of the organics in the V-containing particles. The sea salt factor was composed of a large abundance of ^35^Cl^−^, representing the contribution of sea spray [8,65]. The fraction of each factor in the V-containing particles is shown in Figure 7. The V-containing particles were dominated by the ship emissions factor, which accounted for 35.7%, followed by the dust factor (19.6%) and the industrial emissions factor (17.9%). The ship emissions factor was characterized by EC (^24^C^+^, ^36^C^+^), ^51^V^+^, and sulfate (HSO_4_^−^), suggesting that the freshly emitted ship exhaust particles were aged before arriving at the sampling site [1,2,24,66]. The characteristic ions of the dust factor mainly included ^40^Ca^+^, ^56^CaO^+^, and sulfate.

According to the above analysis, it can be concluded that the V-containing particles mainly originated from three factors (ship emissions factor, dust factor, and industrial emissions factor), and their formation and aging were primarily affected by the ship and industrial emissions factors. Although ship emissions were still a dominant source of the V-containing particles, the contribution of the ship emissions substantially decreased compared with that reported in a study conducted before the implementation of the clean fuel policy [67]. Thus, it should be cautious when using V as a tracer for ship emissions because V from industrial sources and dust sources may interfere with the judgment of the contribution of V from ship sources.

### 3.3. Mixing States of V with Sulfate and Nitrate

The presence of sulfate and nitrate in particles is associated with the aging process and the secondary formation of particles [27,28,40]. In recent years, sulfate and V have been considered to be special tracers for studying ship emissions before the clean fuel policy was implemented [27,28,37,68]. Nitrate is considered to be a marker of particulate aging, and fresh ship emission particles produce a very low nitrate mass spectrometry signal or no nitrate signal, while the nitrate signal in environmental particulate matter is stronger than that of fresh particles, indicating that particulate matter is prone to aging during emission and transport [38,69]. In addition, the increasing abundance of nitrate in fine particles is closely related to haze and O_3_ pollution events in Guangzhou [70]. Thus, the mixing states of V with sulfate and nitrate were investigated in this study to track the aging process of the V-containing particles. Before investigating the sulfate and nitrate in these particles, the general distributions of the sulfate and nitrate in total single particles (particles with and without V) were analyzed to determine the overall characteristics.

**Figure 8:**
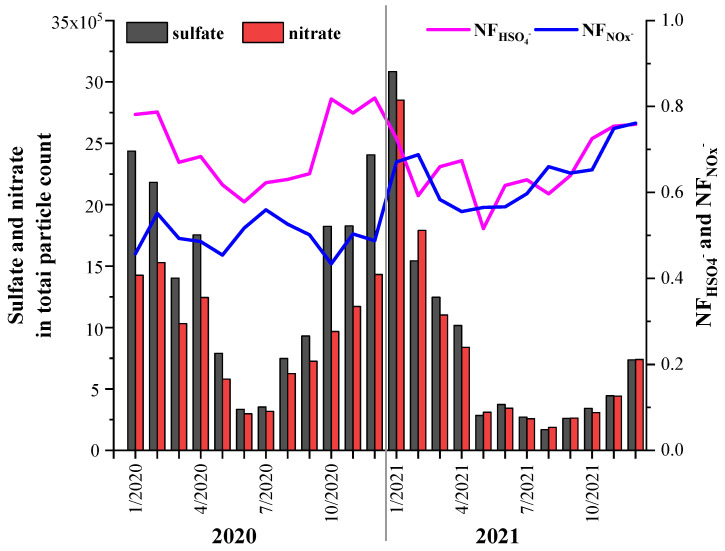
Change trends of sulfate- and nitrate-containing particulate matter and their proportions of the total particulate matter from 2020 to 2021. The bar graph shows the number of particles and the line graph shows the proportions.

The variation trends of the NFs of sulfate and nitrate in the total particles are shown in Figure 8. The particle counts of both sulfate and nitrate were high in the winter and decreased in summer, which was due to changes in their precursors (SO_2_ and NOx) (Figure 3). The particle counts of sulfate were generally higher than those of nitrate in 2020, but they exhibited similar particle counts in 2021. However, their precursors (SO_2_ and NOx) did not exhibit similar trends. Thus, the secondary formation processes of sulfate and nitrate from SO_2_ and NOx may have been different in 2020 and 2021. The NF of sulfate during these two years was 70.3–66.1%, while the NFs of nitrate were 49.7% and 64.2% in 2020 and 2021, respectively. The variation trends of the NFs of sulfate and nitrate were opposite in 2020, but they exhibited similar trends in 2021. Given the identical variation trends of SO_2_ and NOx, the opposite trends of the NFs of sulfate and nitrate may have resulted from their different formation processes. Similar results were also found in the Pearl River Delta region, Hong Kong, and Xiamen [17,18,71].

**Figure 9:**
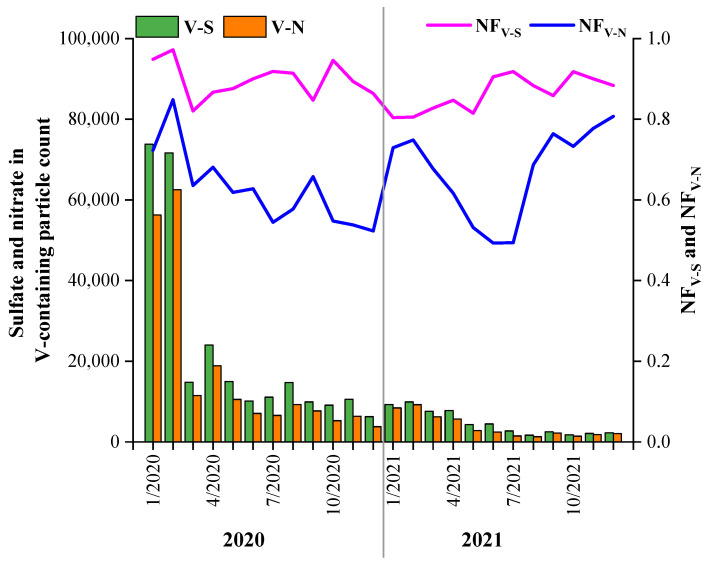
The number and proportion of sulfate and nitrate particulate matter in the V-containing particulate matter in 2020 and 2021. The bar graph shows the quantities and the line graph shows the percentages.

The mixing states of V with sulfate and nitrate were investigated through the variation trends of their NFs in the V-containing particles (Figure 9). The particle counts of the V–sulfate (V–S) and V–nitrate (V–N) particles both decreased from 2020 to 2021, which were different from the particle counts of sulfate and nitrate in the total particles (Figure 8). However, the abundances of sulfate and nitrate in the V-containing particles exhibited trends different from those of their particle counts. The NF of sulfate in the V-containing particles exhibited small peaks in summer and autumn in both years. The increase in sulfate in the V-containing particles was possibly due to the same influence of ship emissions. Zhou et al. (2020) observed that the fraction of sulfate-containing particles exhibited a moderate decrease from 95% in high-sulfur fuel (S_F_) oil (HS) emissions to 78% in low (S_F_) oil (LS) emissions. In this study, the annual average proportions of the sulfate content in the V-containing particles in 2020 and 2021 were 90% and 86%, respectively, which were much higher than that in LS. This can be explained by the fact that the secondary processes in the atmosphere had a strong effect on the V-containing particles. In contrast to sulfate, the NF of the nitrate in the V-containing particles exhibited peaks in winter due to the aged state of the V-containing particles. Since nitrate was not directly emitted from the ship exhaust, the high nitrate content was due to the aging of the V-containing particles. This suggests that the freshly emitted V-containing particles were aged during their transportation from the port to the urban area.

## 4. Conclusions

The annual characteristics and mixing states of V-containing single particles were investigated using SPAMS from 2020 to 2021 in Huangpu Port in Guangzhou. The particle count of the V-containing particles accounted for 1.4% and 0.6% of the total detected single particles in 2020 and 2021, respectively. The decreasing trend of the V-containing particles from 2020 to 2021 may have been due to the implementation of the clean fuel policy and the reduced anthropogenic emissions during the COVID-19 epidemic since 2020. The increase in the NFv of the V-containing particles in summer was attributed to the summer monsoon carrying the air pollutants emitted by ships to the sampling site. The positive matrix factorization analysis revealed that 35.7% of the V-containing particles were from ship emissions in June and July 2020, followed by dust and industrial emissions. Although ship emissions were still a dominant source of the V-containing particles, the contribution of ship emissions substantially decreased compared with the results of studies conducted before the implementation of the clean fuel policy. Thus, care should be taken when using V as a tracer for ship emissions because V from industrial sources and dust sources may interfere with the judgment of V from ship sources. Furthermore, more than 80% of the V-containing particles were found mixing with sulfate and 60% were found mixing with nitrate, suggesting that the majority of the V-containing particles were secondary particles processed during the transport of the ship emissions to the urban area. These results emphasize the significant changes in the distribution of the V-containing particles after the implementation of the clean fuel policy and suggest the reduced role of V as an indicator of ship emissions. It is worth noting that single-particle analysis based on SPAMS only provided semi-quantitative results, which still needs comprehensive field measurements to acquire quantitative results to fully study the annual characteristics of V in atmospheric fine particles. Besides, the detailed source analysis of freshly ship-emitted particles is necessary to explore the authentic distributions of V and other metals to evaluate their changes in the atmosphere after the implementation of the clean fuel policy.

## Data Availability

The observational data, including SPAMS and the meteorological parameters obtained in this study, are available from the corresponding authors upon request (limei2007@163.com).

## Supplementary Materials

The following supporting information can be downloaded at: https://www.mdpi.com/article/10.3390/toxics11040339/s1, Figure S1: Potential source distributions of the V-containing single particles in March, April and May, 2020. Figure S2: Temporal variations of wind speed (WS), wind direction (WD), relative humidity (RH), temperature (T), NO_2_ concentration, SO_2_ concentration, PM_2.5_ concentration, PM_10_ concentration, total ambient particles, V-containing particles and its number fraction (NFv) in total ambient particles from 2020 to 2021 in Guangzhou, China.

## Nomenclature

V	Vanadium
SPAMS	Single-particle aerosol mass spectrometry
EC	Elemental carbon
VOCs	Volatile organic compounds
RPA	Relative peak aera
NF	Number fraction
NFv	Number fraction of V-containing particles
V-S	Sulfate in V-containing particles
V-N	Nitrate in V-containing particles
HS	High-sulfur fuel
LS	Low-sulfur fuel
PMF	Positive matrix factorization
PSCF	Potential source contribution function
$n_{ij}$	Total number of endpoints that fall in the ${ij}_{th}$ cell in PSCF analysis
$m_{ij}$	Number of endpoints of that parcel for which the measured values exceed a user-determined threshold criterion in PSCF analysis
