# Supplementary material for: Long-Term Observation of Mixing States and Sources of Vanadium-Containing Single Particles from 2020 to 2021 in Guangzhou, China
Source: Toxics. 2023 Mar 31;11(4):339. doi: 10.3390/toxics11040339 (PMC10144789; doi:10.3390/toxics11040339)
Supplement: Supplementary file 1 [file toxics-11-00339-s001.zip › toxics-2304988-supplementary.pdf]

Supplementary data

Long-Term Observation of Mixing States and Sources of Vanadium-Containing Single Particles from 2020 to 2021 in Guangzhou, China

Xin Xiong[1], Zaihua Wang[2,3], Chunlei Cheng[1,4,5]*, Mei Li[1,5*], Lijun Yun[1], Sulin Liu[1], Liyuan Mao[1], Zhen Zhou[1,5]

1. Institute of Mass Spectrometry and Atmospheric Environment, Guangdong Provincial Engineering Research Center for Online Source Apportionment System of Air Pollution, Jinan University, Guangzhou 510632, China
2. State Key Laboratory of Organic Geochemistry, Guangzhou Institute of Geochemistry, Chinese Academy of Sciences, Guangzhou 510640, China
3. Institute of Resources Utilization and Rare Earth Development, Guangdong Academy of Sciences, Guangzhou 510650, China
4. State Key Laboratory of Loess and Quaternary Geology, Institute of Earth Environment, Chinese Academy Science, Xi'an 710061, China
5. Guangdong-Hongkong-Macau Joint Laboratory of Collaborative Innovation for Environmental Quality, Guangzhou 510632, China

----

*Correspondence to: Chunlei Cheng (chengcl@jnu.edu.cn) and Mei Li (limei2007@163.com)

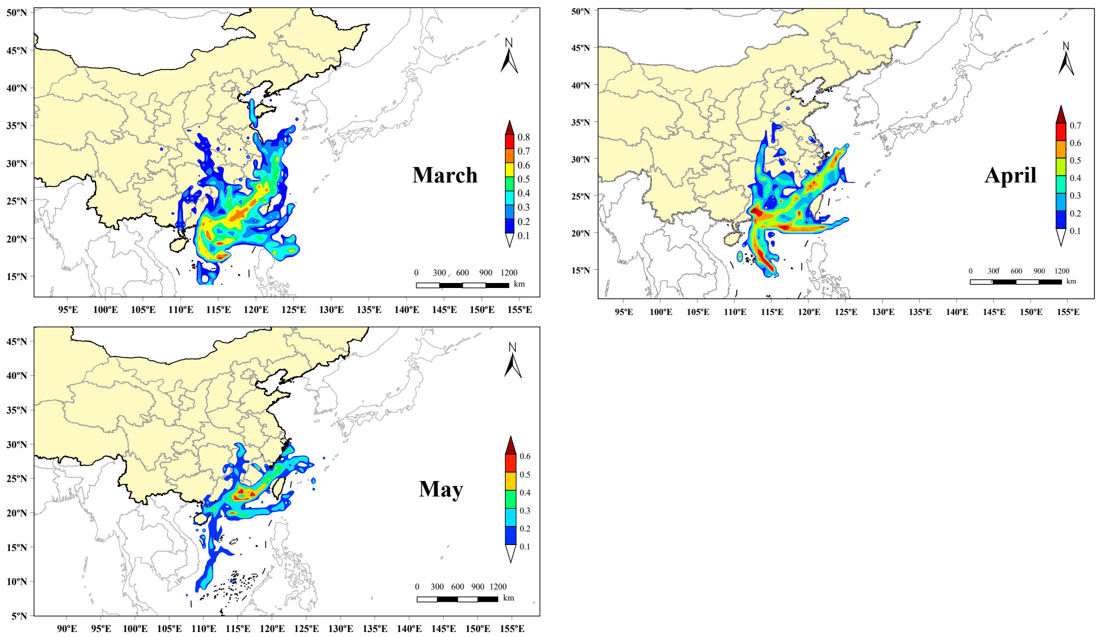

Figure S1. Potential source distributions of the V-containing single particles in March, April and May, 2020. The colors in the legend represent the WPSCF values.

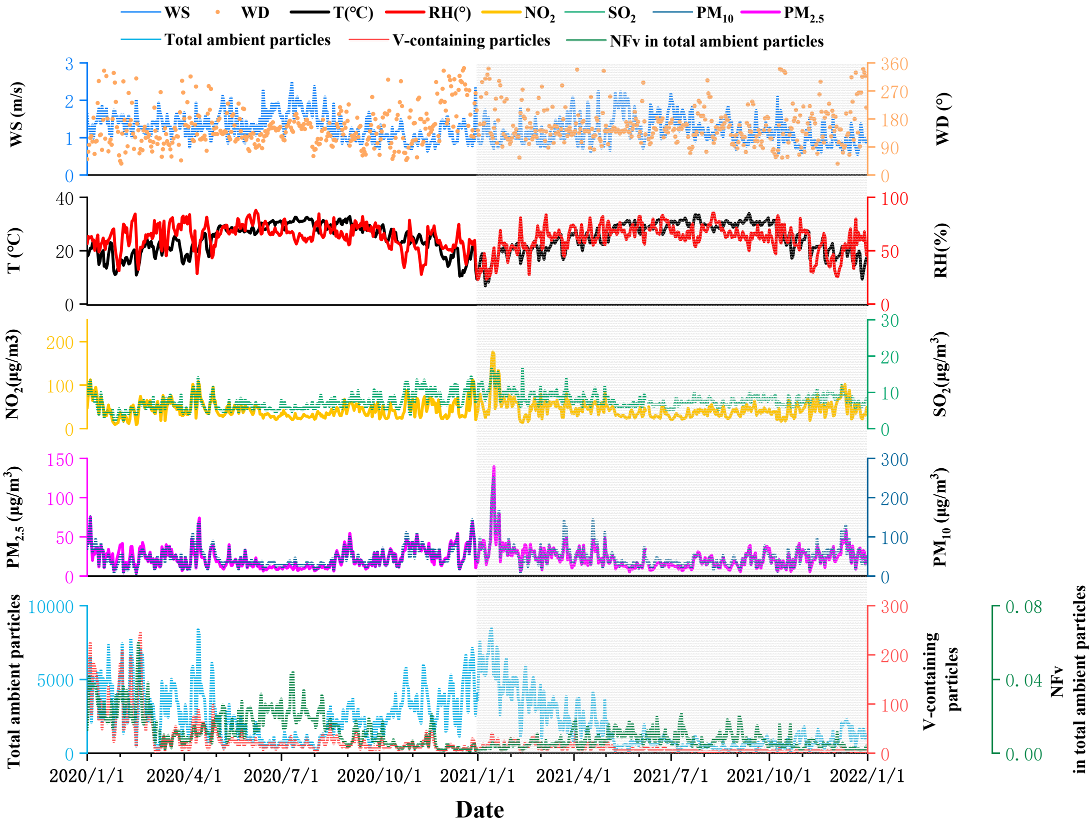

Figure S2. Temporal variations of wind speed (WS), wind direction (WD), relative humidity (RH), temperature (T), NO_2 concentration, SO_2 concentration, $PM_{2.5}$ concentration, PM_{10} concentration, total ambient particles, V-containing particles and its number fraction (NFv) in total ambient particles from 2020 to 2021 in Guangzhou, China. Grey shaded areas represent the data of 2021.
